# Enhancement of curcumin level and hepatoprotective effect in rats through antioxidant activity following modification into nanosized particles

**DOI:** 10.14202/vetworld.2022.2323-2332

**Published:** 2022-09-29

**Authors:** Ni Made Dwi Sandhiutami, Rika Sari Dewi, Sondang Khairani, Raka Nitya Agasti Putri

**Affiliations:** Department of Pharmacology, Faculty of Pharmacy, Pancasila University, Srengseng Sawah, Jagakarsa, Jakarta, Indonesia

**Keywords:** antioxidant, curcumin modified into nanosized particles, hepatoprotective

## Abstract

**Background and Aim:**

Developing curcumin into nanosized particles is one of the approaches to overcome the limited use of curcumin. This study aimed to prepare curcumin into nanosized particles to increase the curcumin level in the rat’s liver and hepatoprotective effect in rats.

**Materials and Methods:**

Curcumin into nanosized particles formulated using ionic gelation method. Rats were divided into four groups (n = 6): Normal, negative, curcumin, and curcumin modified into nanosized particles were treated with 100 mg/kg body weight orally for 14 days. Hepatic curcumin level was investigated using liquid chromatography with tandem mass spectrometry, antioxidant activity by malondialdehyde (MDA), and hepatoprotective effect by aspartate transaminase (AST), alanine transaminase (ALT), and histopathology.

**Results:**

The curcumin level in the rat’s liver in the curcumin group was 12.19 ng/mL, and that in those receiving modified into nanosized curcumin was 209.36 ng/mL. The MDA levels in the normal, negative, curcumin, and curcumin modified into nanosized particles groups were 1.88, 4.87, 3.38, and 1.04 nmol/L, respectively. The AST levels in these groups were 57.12, 130.00, 102.13, and 74.28 IU/L, and the ALT levels were 21.63, 61.97, 39.38, and 28.55 IU/L. The liver histopathology scoring showed that curcumin in nanosized particles was better than curcumin in degeneration of fat, lymphocyte infiltration, and necrosis.

**Conclusion:**

There was a 17 times increase in curcumin level in the liver of rats treated with curcumin modified into nanosized particles. Curcumin modified into nanosized particles showed more significant improvement as antioxidant and hepatoprotector than curcumin.

## Introduction

The liver is the second largest organ and has the most complex functions in the body. It plays a role in the metabolism of food substances as well as most drugs and toxicants. Some substances can undergo detoxification, but many toxicants can reactivate and become more toxic. Hepatocytes are cells that are responsible for the central role of the liver in metabolism [1]. Liver function disorders are still a major health problem in both developed and developing countries. In the United States, research showed that the annual incidence of newly diagnosed chronic liver disease was 72.3% per 100,000 population. Most patients had hepatitis C (57%), followed by alcohol-induced liver disease (24%), nonalcoholic steatosis (9%), and hepatitis B (4%). Riset Kesehatan Dasar (Riskesdas) stated that the prevalence of hepatitis in Indonesia is 2 times higher, from 0.6% in 2007 to 1.2% in 2013 [2, 3]. Liver metabolism can be disrupted due to not only drug consumption but also free radical compounds. Free radicals are molecules that, in their outer orbit, have one or more unpaired electrons, and are very labile and highly reactive, so free radicals play an important role in tissue damage and pathological processes in living organisms. Sources of free radicals are obtained from cigarette smoke, pollutants, X-rays, ozone, vehicle fumes, and chemicals such as carbon tetrachloride (CCl_4_) [4, 5]. The excessive free radical formation will result in oxidative stress, that is an imbalance between the production of oxygen-derived compounds sourced from reactive oxygen species such as superoxide radicals (O_2_^−^), hydroxyl radicals (OH^−^), and peroxyl radicals (RO_2_^−^) with the body’s antioxidant system. The negative impact of oxidative stress in the body is the presence of oxidant attacks on unsaturated fatty acids that cause a chain reaction known as lipid peroxide. This process results in the breakdown of fatty acids into various compounds that are toxic to cells, such as malondialdehyde (MDA). A high level of MDA indicates an oxidation process in the cell membrane [6, 7]. One of these negative impacts is that it can cause disturbances that can damage the liver. Liver damage is characterized by increased levels of MDA, aspartate transaminase (AST), and alanine transaminase (ALT), and histopathological manifestations of liver damage can be in the form of infiltration, fat degeneration, necrosis, and finally, cirrhosis [8, 9]. Judging from the number of cases and indicators of the causes of liver damage that are often found daily, further action is needed, namely, by finding drugs to protect the liver (hepatoprotectors).

Hepatoprotector is a drug compound that can provide protection to the liver from damage caused by drugs, chemical compounds, and viruses. One of the compounds that have been widely developed and used as a hepatoprotector is curcumin [10–15]. Curcumin, bis(4-hydroxy-3-methoxyphenyl)-1,6-diene-3,5-dione, which is also known as diferuloylmethane, is one of the lipophilic phenolic compounds in *Curcuma* *domestica* plant that is widely found in South Asia, India, Indochina, and other Asian countries, including Indonesia. Curcumin is one of the largest components in curcuminoid compounds that are found in the roots of these plants. Curcumin is generally used as a food additive such as a spice or coloring agent and has been widely used in traditional medicine. Many studies have been conducted to determine its benefits. It was found that curcumin has several pharmacological effects, including anti-inflammatory, antioxidant, antiviral, antifungal, antibacterial, immunomodulatory, hepatoprotector, and anticancer [16–18]. Curcumin compounds have pharmacological activity as hepatoprotectors because curcumin has an antioxidant activity that can prevent the formation of free radicals. Curcumin has a phenolic group that can work by capturing and breaking the chain between superoxide ions (O_2_^−^). Curcumin can also work by increasing glutathione S-transferase and inhibiting several inflammatory factors such as nuclear factor-kB and profibrotic cytokines. The content of curcumin compounds also has a quick synergy to increase cholesterol excretion through the intestines so as to prevent and repair fatty liver, as well as regenerate liver cells that are damaged by the effects of chemical toxins [19]. Park *et al*. [20] reported that curcumin at a dose of 100 mg/kg body weight (BW) can reduce the levels of AST and ALT, but the results are less than optimal, which only decreased by 30%. The use of curcumin for treatment is limited due to its low bioavailability [21]. In addition, if used orally, curcumin can undergo first-pass metabolism in the liver and intestines, where curcumin is converted into inactive metabolites. This causes curcumin to be rapidly eliminated in the body, so oral administration of curcumin becomes ineffective [22].

At present, to increase the bioavailability of curcumin, curcumin modified into nanosized particles has been developed [23–25]. In this method, the curcumin preparation was reduced, so that the particle surface area, solubility, dissolution rate, and absorption will increase, increasing the bioavailability of curcumin. Formula development and characterization of curcumin modified into nanosized particles have been conducted using the ionic gelation method. The chitosan polymer used in the ionic gelation method is a polymer that has good mucoadhesive properties so that it can increase drug penetration in the gastrointestinal tract so as to increase the bioavailability of curcumin [26].

This study aimed to test if curcumin made into nanosized particles can increase the level of curcumin in the liver and increase antioxidant activity and hepatoprotective effect.

## Materials and Methods

### Ethical approval

This research was conducted after obtaining ethical approval from the Health Research Ethics Committee of the Faculty of Medicine, Universitas Indonesia (protocol no. KET-63/UN2.F1/ETIK/PPM.00.02/2021). All the actions were taken by minimizing pain and suffering in experimental animals. Rats were acclimatized for 1 week at a temperature of 25°C ± 2°C, humidity of 65% ± 10%, air ventilation of 11–13 times/h, and illumination of 12 h/day (07:00–19:00). Rats were fed with standard pellets and were given *ad libitum* drinking water.

### Study period and location

This study was conducted from August to December 2021. The preparation of curcumin modified into nanosized particles was performed at the Laboratory of Pharmaceutical Technology Formulation of Semisolida, and *in vivo* test was conducted at the Pharmacology Laboratory, Faculty of Pharmacy, Pancasila University, Jakarta, Indonesia.

### Materials

Curcumin (PT. Plamed Green Science Limited, Shaanxi, China; total curcuminoid content, 95%), chitosan, glacial acetic acid, sodium tripolyphosphate (NaTPP), dimethyl sulfoxide (DMSO), 70% ethanol, propylene glycol, Tween 80, glycerin, distilled water, sodium methylcellulose, cremophor (Sigma-Aldrich, St. Louis, MO, USA), Sprague-Dawley rats aged 8 weeks, CCl_4_, trichloroacetic acid (TCA), thiobarbituric acid (TBA) (Sigma-Aldrich), AST and ALT diagnostic kits (Glory-Diagnostic, Montgat, Barcelona, Spain), olive oil, ethylenediaminetetraacetic Acid, Aquadest, and materials for making histopathological preparations (70%, 80%, 90%, and 100% alcohol; 40% formalin; glacial acetic acid; solid paraffin; xylol; 20% hematoxylin; hydrochloric acid), 0.9% NaCl (PT. Widatra Bhakti, Jawa Timur, Indonesia), propranolol (as an internal standard), methyl tert-butyl ether (MTBE; Merck, Darmstadt, Germany), 0.1% formic acid (Merck), acetonitrile (Merck), and methanol (Merck).

### Preparation of curcumin modified into nanosized particles

Curcumin modified into nanosized particles was prepared with a concentration of 1%. One gram of curcumin was mixed with Tween 80 (4 mL), glycerin (10 mL), ethanol (10 mL), propylene glycol (20 mL), DMSO (2 mL), and cremophor (14 mL). Then, 30 mL of 1% chitosan solution was added, mixed, and stirred for 10 min. Subsequently, 10 mL of 0.3% NaTPP was dripped to form nanosized particles. Its manufacture has been developed and characterized in a previous study conducted by Arozal *et al*. [26] with the particle size of modified curcumin was 11.5 nm with PI 0.509, and the zeta potential was +22.78.

### Animal experimental design

In this test, Sprague-Dawley rats were divided into four groups, namely, normal group without any treatment as control, negative group given olive oil, curcumin group given curcumin at a dose of 100 mg/kg BW, and curcumin modified into nanosized particles group given a dose of 100 mg/kg BW, each consisting of six rats. On day 0 (before treatment), blood was collected from each group of rats. All groups, except the normal group, were given the test preparation orally for 14 days, and later on the 14^th^ day, given CCl_4_ 1 mL/kg BW orally. On day 16, rats were euthanized, and blood and liver were collected.

### Serum and tissue isolation

The blood samples were centrifuged at 1100× *g* for 15 min to obtain a clear, slightly yellowish plasma. Plasma was placed in another tube to be checked for levels of MDA, AST, and ALT. The liver was rinsed with 0.9% NaCl and then weighed to calculate the liver weight ratio. The weighed liver was divided into two parts to measure the levels of curcumin in the liver, and the other part was placed into a container containing normal formalin buffer to make histopathological preparations.

### Measurement of curcumin levels in the liver

Curcumin levels were measured using a liquid chromatography with tandem mass spectrometry (LC-MS/MS). The LC-MS/MS system used was ultra-performance liquid chromatography UPLC Waters and tandem mass spectrometry with a positive electrospray ionization source. Validation of the analytical method for determining curcumin levels was according to the European Medicines Agency guideline on bioanalytic method validation [27].

Separation of the analyte in the sample using a liquid chromatography system and a mobile phase gradient in a mixture of 0.1% formic acid and acetonitrile with an analyte rate of 0.3 mL/min and an injection volume of 3 μL. The optimal mobile phase composition used was 0.1% formic acid and acetonitrile, with a ratio of 72:28.

Twenty microliters of internal standard and 2 mL of MTBE were added to 200 μL of liver homogenate, then vortexed for 1 min, and centrifuged at 1500× *g* for 10 min, and the supernatant was collected. The supernatant was evaporated using an evaporator with nitrogen gas flowing at 60°C for 2 min. The residue was reconstituted with 100 μL of mobile phase and vortexed for 30 s. The solution was centrifuged at 6000× *g* for 5 min at 20°C and prepared for analysis using LC-MS/MS [28].

### Measurement of MDA level

Plasma MDA levels were measured according to the Wills method. A total of 200 μL of plasma was added with 1.0 mL of 20% TCA and 2 mL of 0.67% TBA. The solution was homogenized, heated in a water bath for 10 min, and then cooled. After cooling, the solution was centrifuged at 1500× *g* for 10 min, and the filtrate was collected. The pink filtrate was measured absorption with an ultraviolet-visible spectrophotometer (Genesys, Thermo Scientific, USA) at a wavelength of 532 nm. Malondialdehyde levels were calculated using the standard MDA curve with a concentration of 0, 0.0005, 0.0010, 0.0020, 0.0040, 0.0080, 0.0160, 0.0320, and 0.0640 nmol [7].

### Measurement of AST level

One hundred microliters of the sample reacted with 1000 μL of monoreagent (800 μL R1 (121 mmol/L TRIS pH 7.8, 362 mmol/L l-aspartate, >460 U/L MDH, >600 U/L lactate dehydrogenase [LDH]) and 200 μL R2 (75 mmol/L 2-oxoglutarate, 1.3 mmol/L NADH)). Read the AST levels listed on the microlab 300.

U/L at a wavelength of 340 nm = ΔA/min × factor = ΔA/min × 1745.

### Measurement of ALT level

One hundred microliters of the sample reacted with 1000 μL of monoreagent (800 μL R1 (150 mmol/L TRIS pH 7.3, 750 mmol/L l-alanine; >1,350 U/L LDH) and 200 μL R2 (75 mmol/L 2-oxoglutarate, 1.3 mmol/L NADH)). Read the ALT levels listed on the microlab 300.

U/L at a wavelength of 340 nm = ΔA/min × factor = ΔA/min × 1745.

### Histopathological examination of the rat’s liver

The liver was fixed in a 10% formalin buffer overnight (12 h), and tissue sections were carefully dissected from representative areas following the guidelines of the grossing technique and processed in the automated tissue. The selected tissue was processed with the principle of dehydration, clearing, and embedding on an automatic device. The tissue was embedded in paraffin blocks and then cut into thin slices using a rotatory microtome with a thickness of 5 μM. The slices were immersed in 50°C hot water and then transferred to a microscope glass slide to be stained with hematoxylin and eosin (HE). The sections stained with routine HE staining were viewed under a light microscope.

Observations were made by comparing the histological preparations between the liver of the control group and the liver of the treatment group. Assessment of liver lobular damage was done by giving a score or score on liver lobular damage using a binocular light microscope at 400× (Leica DM500, Danaher, Germany). Value or damage was divided into four levels according to the classification of Mordue *et al*. [29]: 0, no damage occurred; 1, 1%–20% damage; 2, 21%–50% damage; 3, 51%–75% damage; and 4, >75% damage.

### Statistical analysis

Data were analyzed using Statistical Package for the Social Sciences 20 program. Data on levels of MDA, AST, and ALT obtained on day 16 were tested for normality and homogeneity. If the data were normally distributed and homogeneous, the data were analyzed by parametric statistics using a one-way analysis of variance method (p < 0.05). The histopathological scoring results were ordinal data with four levels of damage: Score 0 if one field of view has no degeneration, lymphocytic infiltration, and necrosis in the observed area; Score 1 if one field of view has 1%–20% degeneration, lymphocytic infiltration, and necrosis in the observed area; Score 3 if one field of view has 51%–75% degeneration, lymphocytic infiltration, and necrosis in the observed area; and Score 4 if one field of view has >75% degeneration, lymphocytic infiltration, and necrosis in the observed area. The scoring value was tested by Kruskal–Wallis nonparametric statistics to determine the difference between each group and continued with the Mann–Whitney test.

## Results

In the present study, the preparation of curcumin modified into nanosized particles was conducted several times. Measurement of curcumin levels was performed in each manufacture of curcumin modified into nanosized particles. The results obtained in this test are the level of curcumin in each manufacturer was around 1%. The consistency of making curcumin modified into nanosized particles is shown in Table-1.

**Table-1:** Results of measuring curcumin levels in curcumin modified into nanosized preparations.

Sample	Curcumin concentration (%)
1	1.035
2	0.996
3	1.018
x̅± SD	1.007 ± 0.016

**Figure-1:**
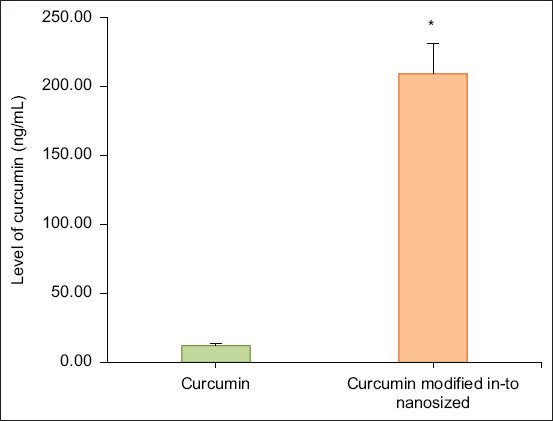
Increase curcumin level in rat Hepar after given curcumin modified in-to nanosized at dose of 100 mg/kg body weight for 14 days. (*p < 0.05 vs. curcumin group).

Measurement of curcumin levels in the liver was performed to determine the difference in curcumin reaching the liver between rats receiving curcumin and rats receiving curcumin modified into nanosized particles. The level of curcumin in the liver of rats that received curcumin was 12.19 ± 1.43 ng/mL, and the level of curcumin in the liver of rats that received curcumin modified into nanosized particles was 209.36 ± 22.11 ng/mL. There was an increase in curcumin level of 17× in the liver of rats that received curcumin modified into nanosized particles. The measurement results are shown in Figure-1.

### *In vivo* antioxidant effects of curcumin and curcumin modified into nanosized particles on MDA levels and hepatoprotective effects on AST and ALT levels

**Figure-2:**
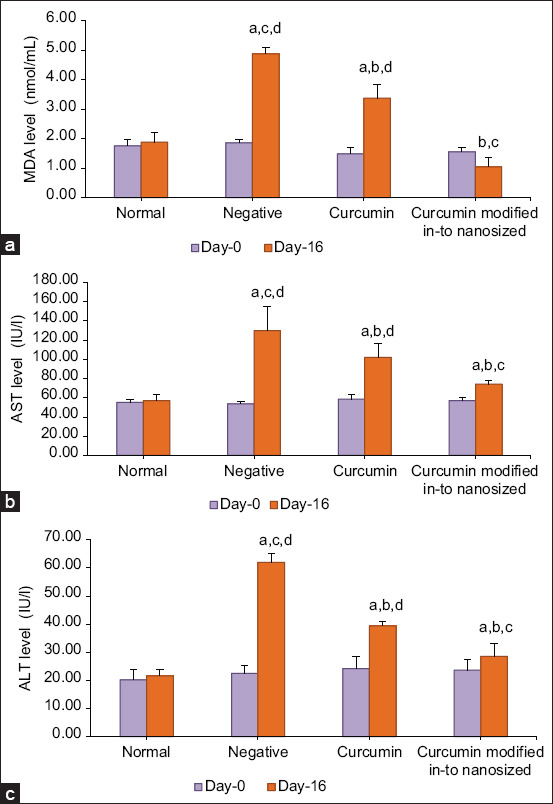
(a) Decreased malondialdehyde concentration (nmol/mL); (b) decreased aspartate transaminase concentration (IU/l); (c) decreased alanine transaminase concentration (IU/l) in Hepar rat after given curcumin modified in-to nanosized at dose of 100 mg/kg body weight for 14 days. Data in mean ± standard deviation (a) p < 0.05 versus normal group, (b) ep < 0.05 versus negative group, (c) p <0.05 versus curcumin group, (d) nilai p < 0.05 versus curcumin modified in-to nanosized group.

The results of measuring MDA levels on days 0 and 16 using a spectrophotometer using the Wills method in each experimental animal group are presented in Figure-2a. Aspartate transaminase levels on days 0 and 16 using microlab 300 in each experimental animal group are presented in Figure-2b. Alanine transaminase levels on days 0 and 16 in each group of experimental animals are presented in Figure-2c.

### Liver weight to rat BW ratio

Liver weight ratio data were calculated by comparing liver weight with rat BW on day 16. The results of the analysis showed that there was a significant difference between the negative group and the curcumin group and the curcumin modified into nanosized particles group. The liver weight ratio was lower in rats in the treatment groups (curcumin and curcumin modified into nanosized particles) than in the negative group. The results of the calculation of the liver weight ratio are shown in Table-2.

**Table-2:** Heart weight ratio.

Group	Rat weight (g)	Heart weight (g)	Heart weight ratio
Normal	165.67 ± 6.56	7.03 ± 0.24	0.0446 ± 0.002
Negative	147.5 ± 12.06	8.91 ± 0.56	0.0607 ± 0.004^a,c,d^
Curcumin	153.5 ± 6.89	8.37 ± 0.14	0.0546 ± 0.002^a,b^
Curcumin modified into nanosized	156.67 ± 7.06	7.59 ± 0.36	0.0484 ± 0.003^a,b,c^

^a^ p<0.05 versus normal group, ^b^ p<0.05 versus negative group, ^c^ p<0.05 versus curcumin group, ^d^ p<0.05 versus curcumin modified into nanosized group. p*<*0.05 after performing the one-way Kruskal-Wallis test followed by the Mann–Whitney test

### Histopathology of rat liver by assessing fat degeneration, leukocyte infiltration, and necrosis

Fatty degeneration or steatosis is a pathological picture characterized by fat accumulation in liver cells caused by free radicals from CCl_4_. Lymphocyte infiltration is the accumulation of large numbers of lymphocytes in the tissue. Necrosis is the death of cells or tissues in living organisms. Statistical analysis showed that the negative group had a significant difference from the treatment group, namely, the curcumin and curcumin modified into nanosized particles groups.

**Figure-3:**
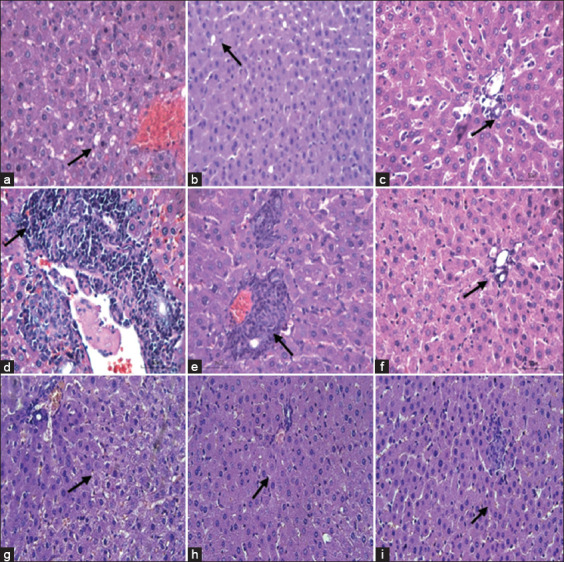
Representative histopathology profiles of the rat Hepar. (a) Negative group, hepatocyte cells that undergo fat degeneration are characterized by cytoplasmic vacuolization. (b and c) Curcumin and curcumin modified into nanosized can reduce fat degeneration caused by CCl_4_. Curcumin modified into nanosized has better effectiveness than curcumin. (d) Negative group, liver damage in the form of lymphocyte infiltration caused by CCl_4_. (e and f) Curcumin and curcumin modified into nanosized have the ability to regenerate hepatocyte cells that are damaged by lymphocytic infiltration. (g) Negative group necrosis in liver cells with changes in the cytoplasm and cell nucleus. (h and i) Curcumin and curcumin modified into nanosized regeneration process occurs from necrosis. In the curcumin group (H), there were still hepatocytes that were not neatly arranged and there were still hepatocytes that were not round in shape, but the modified in-to nanosized curcumin group began to look neat and arranged in a radial manner. H and E staining, magnification at 400×.

Representative histopathology profiles of fat degeneration, leukocyte infiltration, and necrosis of the rat liver are presented in Figure-3. The results of scoring fat degeneration, lymphocyte infiltration, and necrosis in each experimental animal group are presented in Table-3.

**Table-3:** Fat degeneration score results.

Group	Fat degeneration	Lymphocyte infiltration	Necrosis
Normal	0	0	0
Negative	2.63 ± 1.02^a,c,d^	2.67 ± 1.01^a,c,d^	2.87 ± 0.86^a,c,d^
curcumin	1.13 ± 0.71^a,b^	1.13 ± 0.16^a,b,d^	1.03 ± 0.92^a,b^
Curcumin modified into nanosized	0.43 ± 0.67^b^	0.50 ± 0.55^b,c^	0.27 ± 0.41^b^

^a^ p<0.05 versus normal group, ^b^ p<0.05 versus negative group, ^c^ p<0.05 versus curcumin group, ^d^ p<0.05 versus curcumin modified into nanosized group. p*<*0.05 after performing the one-way Kruskal–Wallis test followed by the Mann–Whitney test

## Discussion

Curcumin has low bioavailability; this can be caused by low absorption of curcumin, high metabolism, and fast elimination from the body [30]. The low bioavailability of curcumin has been seen in several animal and human studies [31]. Other studies have shown that the presence of a mucus barrier consisting of glycoproteins, water, and lipids can hinder its diffusion from the lumen to the surface of enterocytes. Mucus can bind curcumin nonspecifically. Berginc *et al*. [32] reported that mucus removal using N-acetyl cysteine in rat jejunum can increase curcumin absorption. The permeability of curcumin to the membrane also depends on the pH where the permeability increases under acidic conditions. Several attempts have been made to increase the absorption of curcumin into nanosized curcumin [33].

Absorbed curcumin can be distributed to the liver and kidneys. In addition, curcumin is distributed in the spleen, lungs, heart, bones, and brain [33, 34]. However, the low level of curcumin in plasma is caused by the high metabolism of curcumin in the body. Curcumin undergoes phases I and II of metabolism. In phase I, curcumin was reduced to tetrahydrocurcumin, hexahydrocurcumin, dihydrocurcumin, and hexahydrocurcumin. In phase II, curcumin and/or its metabolites are conjugated with monoglucuronide or monosulfate catalyzed by b-glucuronidase enzymes. The metabolites formed include curcumin glucuronide and curcumin sulfate, which are found in the urine on oral administration of curcumin. Dihydrocurcumin glucuronide, tetrahydrocurcumin glucuronide, and hexahydrocurcumin are found in the bile [30, 35, 36]. Pandey *et al*. [37] found that most of the metabolites of curcumin are found in feces during oral and intraperitoneal administration.

Pharmacokinetics of a substance or drug can be improved by formulation and particle size reduction. Reducing the particle size will increase the surface area of the particles. Decreasing the particle size below 1 μM will increase the solubility [38]. The nanosized particle size is useful in modeling drug delivery to reach and act on target organs. Drugs using nanoparticle technology are generally drugs with low oral solubility and bioavailability [24, 39]. Into nanosized particles can increase absorption, plasma levels, and AUC values.

The manufacture of curcumin into nanosized particles is also useful for the release of modified drugs for the reasons of increasing bioavailability, avoiding the first metabolic pathway through the liver, protecting tissues in the gastrointestinal tract for irritating drugs, increasing drug stability, and avoiding damage to active substances from certain enzymes [39].

The nanosized form of curcumin in this study can increase curcumin levels in the liver after being given orally to mice. Repeated administration for 14 days in experimental animals with a hepatotoxic model also showed differences in curcumin levels in the group receiving nanosized curcumin and the group receiving curcumin. This can provide information that nanosized curcumin can reach target organs better to be utilized *in vivo* for hepatotoxicity. The level of curcumin in the liver of rats that received curcumin was 12.19 ± 1.43 ng/mL, and the level of curcumin in the liver of rats that received curcumin modified into nanosized particles was 209.36 ± 22.11 ng/mL. There was an increase in curcumin level of 17× in the liver of rats that received curcumin modified into nanosized particles.

Curcumin has several pharmacological effects, including antioxidant, anti-inflammatory, antifungal, antibacterial, and anticancer [16]. As an antioxidant compound, curcumin can reduce or prevent cell damage. In general, antioxidants are compounds that can delay, slow down, and prevent the oxidation processes in living cells or, specifically, are substances that can prevent free radical reactions in lipid oxidation. Physiologically, free radicals are produced in the body and neutralized by endogenous antioxidants, such as catalase and superoxide dismutase (SOD). However, if the production of free radicals is not balanced with endogenous antioxidant activity, a condition known as oxidative stress will arise. This situation can cause cell damage, accelerate the aging process, and cause various diseases such as heart disease, diabetes mellitus, hepatotoxicity, and cancer. The antioxidant activity of curcumin was obtained from the phenolic OH group, which was indicated by the inhibition of lipid peroxidation and the scavenger of free radicals 1,1-diphenyl-2-picryl hydrazyl and nitric oxide radicals [40]. The anti-inflammatory activity of curcumin is shown through the inhibition of several compounds involved in inflammation, such as phospholipase, lipoxygenase, and cyclooxygenase [41]. Curcumin also suppresses cyclooxygenase-2, a pro-inflammatory cytokine, and tumor necrosis factor-α expression and release [42]. Therefore, curcumin also has an effect as a hepatoprotector that can suppress the inflammatory process that occurs in hepatotoxins.

The hepatoprotective mechanism occurs because of the effect of curcumin as an antioxidant that is able to capture superoxide ions and break the chain between superoxide ions (O_2_^−^), thereby preventing liver cell damage due to lipid peroxidation mediated by an antioxidant enzyme, namely, SOD, where the SOD enzyme will convert O_2_^−^ into a less toxic product [19]. The phenol group in curcumin plays a role and has acted as a hepatoprotector. Phenolic groups act as antioxidants which can bind oxygen so that oxygen is not available for the oxidation process; besides that, it can also bind metals that are able to catalyze oxidation reactions [13].

CCl_4_ is a xenobiotic that induces lipid peroxidation and liver damage, so it is often used in testing the hepatoprotector activity of a substance. A single dose of 1.0 mL/kg BW CCl_4_ in male rats can cause liver damage. In the endoplasmic reticulum of the liver, CCl_4_ is metabolized by cytochrome P4502E1 into trichloromethyl free radicals (CCl_3_) 1,2-trichloromethyl with oxygen to form trichloromethylperoxy radicals that can attack endoplasmic reticulum membrane lipids at a rate that exceeds trichloromethyl free radicals. Furthermore, trichloromethylperoxy causes lipid peroxidation, thereby disrupting Ca^2+^ homeostasis and ultimately causing cell death [5]. The increase in lipid peroxidation by free radicals can be assessed by many methods, including the measurement of the primary or secondary products of lipid peroxidation. The primary products of lipid peroxidation include conjugated dienes and lipid hydroperoxides, and the secondary product includes MDA [43]. Liver damage is characterized by elevated levels of the enzymes ALT and AST [5].

The current study showed an increase in the average plasma MDA level in the negative control group. An increase in MDA levels indicates an increase in lipid peroxidation that proves that oxidative stress occurs due to the administration of the chemical compound CCl_4_. The treatment group that was given curcumin modified into nanosized particles had the lowest MDA levels compared to the group that was given curcumin. This is because the bioavailability of curcumin modified into nanosized particles has been increased so that it is absorbed in the body and reaches target organs better and can reduce plasma MDA levels more.

In the present study, the AST level in the normal group was 57.12 IU/L, and the ALT level in the normal group was 21.63 IU/L. These results are consistent with the literature that states that normal AST levels in rats are 45.7–80.8 IU/L and normal ALT levels in rats are 17.5–30.2 IU/L [44]. The results of the analysis showed that the levels of AST and ALT on day 16 in the negative group were significantly different from the curcumin group and the curcumin modified into nanosized particles group. The average AST and ALT levels on day 16 in the negative group were the highest compared to all groups, namely, an increase of 2–3× from normal levels that indicated liver damage. This is caused by the administration of a hepatotoxicant, namely, CCl_4_, where radicals from CCl_4_ can cause damage to hepatocytes resulting in changes in permeability that cause cell contents to come out marked by increased levels of AST and ALT [9]. The statistical results of the treatment group showed that there were significant differences in AST and ALT levels between the curcumin and curcumin modified into nanosized particles groups. Curcumin modified into nanosized particles at a dose of 100 mg/kg BW has a better effectiveness than curcumin at a dose of 100 mg/kg BW in reducing AST and ALT levels; this may be due to the slow absorption of curcumin in the body when given orally. Thus, curcumin modified into nanosized particles can be better at reducing and inhibiting the formation of free radicals so that AST levels are lower.

The ratio of liver weight is one of the parameters that can be used to determine the presence of toxicity that occurs in the liver. In conditions of toxicity to the liver, the weight of the liver will increase in proportion to the toxicity that occurs in the liver. In rats exposed to CCl_4_, the liver weight ratio was greater than the normal condition indicating the occurrence of liver swelling due to damage to liver cells. Damage to liver cells results in a large number of fragments of damaged cells and organelles causing inflammation or fluid mass buildup, which, in turn, causes a buildup of white blood cells in the area responsible for phagocytosis. In the present study, the mean liver weight ratio in the negative group was the highest compared to all groups. In rats in the treatment group (curcumin and curcumin modified into nanosized particles), the liver weight ratio was lower than in the negative group. This indicates that there has been attenuation or inhibition of free radicals formed in the treatment group that cause damage to liver cells. Not as severe as the negative control group. Curcumin modified into nanosized particles at a dose of 100 mg/kg BW has better effectiveness than curcumin at a dose of 100 mg/kg BW; this is because curcumin is absorbed slowly in the body when given orally. Hence, that modified into nanosized curcumin can be better at reducing and inhibiting the formation of free radicals which can prevent hepatocyte damage that can cause inflammation and buildup of fluid mass so that the liver weight ratio is low. The liver weight ratio in this study is directly proportional to the research conducted by Wikanta *et al*. [45], who stated that the greater the weight of the liver, the greater the occurrence of damage to the liver.

CCl_4_ causes lipid peroxidation so that it will disrupt Ca^+^ homeostasis and cause fatty liver cells [5]. Fat degeneration can also be caused by failure of triglyceride transport from hepatocyte cells due to decreased apolipoprotein synthesis, failure of triglyceride binding, and failure of lipoprotein transport across cell membranes. In addition, the mobilization of triglyceride stores in peripheral tissues supports the occurrence of fat [46].

Hepatocyte cells that undergo fat degeneration are characterized by cytoplasmic vacuolization. Normal hepatocytes, namely, hepatocytes, are round, are radially arranged, and have clear cytoplasm, and it was seen in the normal group of hepatocytes in this study. The negative group had the highest average fat degeneration score among all groups. This is because the negative group was given CCl_4_ hepatocytes without prior treatment, so they could not prevent lipid peroxidation that could lead to an increase in fat degeneration. This is also shown in the research conducted by Arafah *et al*. [47]. In the current study, the negative group had the highest score compared to the other groups, due to the induction of CCl_4_. Furthermore, in this study, the curcuminoid group had the lowest infiltration score compared to other groups. Induction of CCl_4_ causes an oxidation chain reaction that can damage the structure of liver cells. Damaged cell structures can increase the migration and phagocytosis of inflammatory cells and macrophages from blood vessels to damaged tissues. On the other hand, phagocytosis increases free radicals in the cells and surrounding tissues because phagocytosis produces free radicals by inflammatory cells and macrophages. This results in a chain reaction of free radicals that can damage surrounding cells that are still healthy. Inflammation will activate the Kupffer cells and monocyte migration from the blood circulation and result in an increase in the number of macrophages [47].

The occurrence of necrosis in liver cells can be identified by changes in the cytoplasm and the nucleus of the cells. When the plasma membrane of liver cells is damaged, various enzymes and cytosol are released into the blood and can be used as a marker of the extent and type of liver cell damage. Microscopically, hepatocytes have a round nucleus, clear cytoplasm, and are arranged regularly to form a plate that radiates to the center of the lobules. In the present study, the microscopic picture of normal group hepatocytes showed this. The administration of hepatotoxicant substances, namely, CCl_4_, causes the formation of free radicals and eventually severe cell damage will occur. Microscopically, it was also clearly seen in the negative group that the hepatocyte cells were not neatly arranged and the hepatocytes were not round.

The statistical results of scoring fat degeneration, leukocyte infiltration, and necrosis between the negative group and the treatment group, namely, the curcumin group and curcumin modified into nanosized particles, were significantly different. This shows that the curcumin and curcumin modified into nanosized particles groups with a dose of 100 mg/kg BW each have been able to reduce liver damage in the form of fat degeneration, leukocyte infiltration, and necrosis caused by CCl_4_; this can be seen from the decrease in scores in the curcumin group and nanocurcumin.

In the present study, fat degeneration, leukocyte infiltration, and necrosis in the curcumin and curcumin modified into nanosized particles groups showed significant differences. When viewed visually, there was more fat degeneration in in the curcumin group than in the curcumin modified into nanosized particles group. Curcumin modified into nanosized particles has better effectiveness in regenerating hepatocyte cells than curcumin. This is because curcumin has low bioavailability and is eliminated faster than curcumin modified into nanosized particles when administered orally. When compared with the normal group, curcumin modified into nanosized particles has the same effectiveness.

The results of scoring degeneration, leukocyte infiltration, and necrosis in this study were not in accordance with the research conducted by Hadisoewignyo *et al*. [48], where the curcumin group had a lower score than the curcumin modified into nanosized particles group, although it was not statistically significant. In this study, it was concluded that nanocurcumin with mesoporous silica nanoparticles carriers had been widely reported to cause toxicity. In this study, curcumin modified into nanosized particles made by ionic gelation using chitosan polymer has a lower score than curcumin. This is in accordance with the theory that explains that the nanoparticle form will increase the bioavailability of curcumin so that it can be better as a hepatoprotector in preventing liver damage. In this case liver damage in the form of degeneration, leukocyte infiltration, and necrosis.

Curcumin and nanocurcumin have the potential to protect the liver and can perform the regeneration process from damage in the form of necrosis. Microscopically, it looks different, where in the curcumin group, there are still hepatocytes that are slightly not neatly arranged, and there are still hepatocytes that are not round but not as bad as the negative group. In the nanocurcumin group, it was clear that the arrangement of hepatocytes began to look neat, and that overall, they were arranged in a radial manner.

## Conclusion

Curcumin modified into nanosized particles can enhance the curcumin level in the liver by 17× and can reduce CCl_4_ hepatotoxicity by decreasing MDA, AST, and ALT levels more effectively than curcumin. Curcumin nanoparticles can repair liver damage better than curcumin by improving fat degeneration, lymphocyte infiltration, and necrosis of the liver.

## Authors’ Contributions

NMDS: Contributed to conceptual design, supervised the experiments, and drafted the manuscript. RSD: Conducted the *in vivo* experiments, data analysis, and wrote the manuscript. SK: Conducted the serum and plasma assessment, data analysis, and revised the manuscript. RNAP: Conducted the experiment, histopathological analysis, and performed data analysis. All authors have read and approved the final manuscript.
